# mRNA vaccines: a new opportunity for malaria, tuberculosis and HIV

**DOI:** 10.3389/fimmu.2023.1172691

**Published:** 2023-04-24

**Authors:** Laura Matarazzo, Paulo J. G. Bettencourt

**Affiliations:** ^1^ Center for Interdisciplinary Research in Health, Universidade Católica Portuguesa, Lisboa, Portugal; ^2^ Faculty of Medicine, Universidade Católica Portuguesa, Rio de Mouro, Portugal

**Keywords:** RNA vaccines, malaria, tuberculosis, HIV, infectious diseases

## Abstract

The success of the first licensed mRNA-based vaccines against COVID-19 has created a widespread interest on mRNA technology for vaccinology. As expected, the number of mRNA vaccines in preclinical and clinical development increased exponentially since 2020, including numerous improvements in mRNA formulation design, delivery methods and manufacturing processes. However, the technology faces challenges such as the cost of raw materials, the lack of standardization, and delivery optimization. MRNA technology may provide a solution to some of the emerging infectious diseases as well as the deadliest hard-to-treat infectious diseases malaria, tuberculosis, and human immunodeficiency virus/acquired immunodeficiency syndrome (HIV/AIDS), for which an effective vaccine, easily deployable to endemic areas is urgently needed. In this review, we discuss the functional structure, design, manufacturing processes and delivery methods of mRNA vaccines. We provide an up-to-date overview of the preclinical and clinical development of mRNA vaccines against infectious diseases, and discuss the immunogenicity, efficacy and correlates of protection of mRNA vaccines, with particular focus on research and development of mRNA vaccines against malaria, tuberculosis and HIV.

## Introduction

From historical empirical immunization methods or “variolation” against smallpox, and the development of the first live-attenuated whole pathogen vaccines in early 1900’s, the field of vaccinology has been constantly improving. While live-attenuated vaccines are still widely used, the current arsenal of vaccines includes killed whole organisms (inactivated vaccines), toxoid, subunit vaccines (purified proteins and peptides, polysaccharides), conjugated vaccines (protein-polysaccharide conjugate), virus-like particles, outer membrane vesicles, viral vectored, and nucleic acid vaccines, including mRNA vaccines (1, 2).

Vaccination is undoubtedly amongst the greatest advances of medicine, preventing millions of deaths to infectious diseases each year. National and international vaccination programs and mass vaccination campaigns have been responsible for a significant decrease in mortality, particularly during childhood (3). Importantly, vaccination led to the complete eradication of smallpox (4). However, despite this success, current technologies have failed to provide truly effective protection against human immunodeficiency virus (HIV), tuberculosis (TB) and malaria, which remain among the main causes of death, particularly in low and middle-income countries (LMIC). Indeed, the only available vaccine against TB, a live-attenuated strain of *Mycobacterium bovis*, bacillus Calmette-Guérin (BCG), has inconsistent efficacy, does not prevent transmission, and no vaccine against HIV or Malaria has been licensed to date.

The recent COVID-19 pandemic and the quick development of mRNA-based vaccines have drawn attention to this technology. These vaccines are safe, efficient, rapid and relatively simple to produce, and may quickly respond to the needs in emergency settings such as a pandemic caused by emerging pathogens (5). The research on mRNA vaccines has grown exponentially, and vaccine candidates against a great variety of infections have entered clinical trials, including HIV, TB and malaria. This brings hope that mRNA technologies could offer a solution to prevent these deadly diseases, but also for other applications such as cancer treatment and protein-replacement therapies for genetic disorders (6).

mRNA vaccine technology presents several advantages compared to other types of vaccines. They are safer and generally well-tolerated in healthy patients. Live attenuated vaccines, present a risk of uncontrolled replication in immunocompromised subjects, which does not occur with mRNA vaccines. Inactivated vaccines induce essentially humoral response, while mRNA vaccines activate both humoral and cellular responses. In addition, unlike recombinant protein-based vaccines which often need adjuvants to improve their immunogenicity, mRNA vaccine has intrinsic adjuvant activity (5). Furthermore, mRNA *in vitro* synthesis and purification processes are cell-free, quick and easily scalable, in contrast with the fastidious and time-consuming production of conventional vaccines (7).

In this review we focus on the mRNA vaccine formulation design, delivery methods and manufacturing processes. We describe an overview on the preclinical and clinical development of mRNA vaccines against infectious diseases, and we discuss their immunogenicity, efficacy, and correlates of protection, with particular focus on the three major killers, malaria, TB and HIV.

## mRNA vaccine design, production and optimization

### Structure and biological function

Messenger RNA (mRNA) is a single strand nucleic acid molecule encoding genetic information of one or several genes. Endogenous mRNA is transcribed in the nucleus of cells from genomic DNA by the RNA polymerase, either constitutively or under the control of transcription factors that activate or repress gene expression. mRNA is then transported to the cytoplasm, where it is translated by the ribosomal translation machinery into the encoded protein.

**Figure 1:**
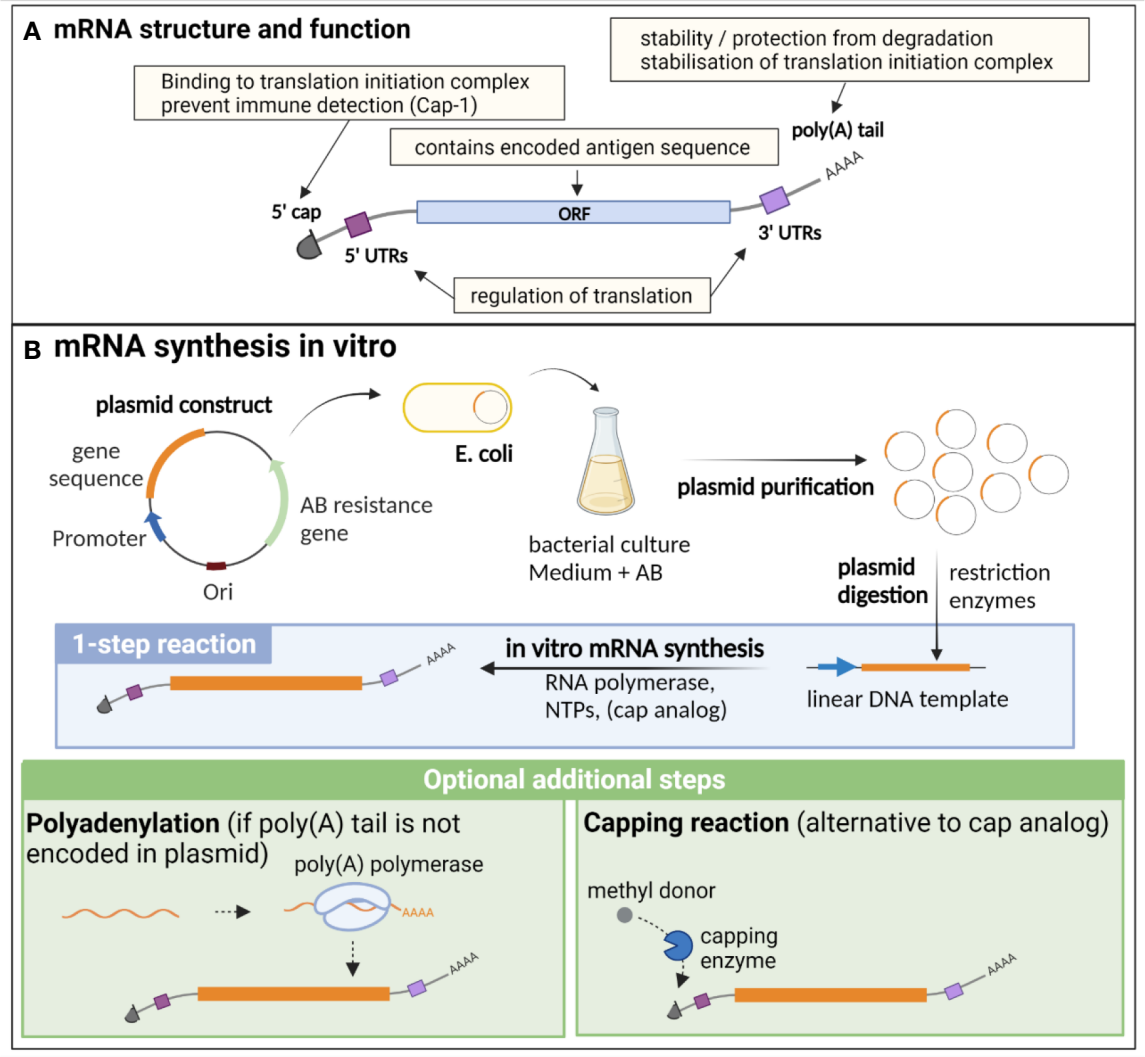
Structure, function and *in vitro* synthesis of vaccine mRNA. **(A)** mRNA are single stranded nucleic acids composed of an open reading frame (ORF) encoding the gene of interest, flanked by untranslated regions (UTRs) implicated in translation regulation, a cap at the 5’ end consisting of a N7-methylated guanosine residue, important for translation initiation and immune detection, and a poly(A) tail at the 3’ end, participating in the stability of the mRNA, as well as the stabilisation of the translation initiation complex. **(B)** *In vitro* synthesis of mRNA is often performed from a linearised plasmid template. The gene of interest is encoded in the plasmid template downstream of a promoter sequence. *E. coli* are transformed with the plasmid and cultured in liquid medium containing an antibiotic for which the plasmid encodes a resistance gene, thereby allowing the selection of bacteria that express the plasmid. The plasmid is then purified from the culture and digested using restriction enzymes to obtain a linear DNA template. *In vitro* transcription of mRNA is performed in the presence of the DNA template, an RNA polymerase and nucleotides triphosphates (NTPs). The capping can be performed by directly adding a cap analogue in the IVT reaction mix (1-step reaction), or alternatively by an enzymatic capping reaction after the IVT. If the poly(A) tail is not encoded in the plasmid, an additional step of polyadenylation is required. Created with BioRender.com.

Endogenous eucaryotic mRNA is composed of an open reading frame (ORF), which contains the sequence of the encoded genes. This ORF is flanked by 5’ and 3’ untranslated regions (UTRs), that are important for the regulation of translation, as well as for the stability of the molecule. Additional essential structures are the cap, consisting of a N7-methylated guanosine residue (m (7)Gppp) at the 5’ end, and the poly(A) tail at the 3’ end, composed of approximately 250 to 300 adenosine residues (5, 8) (**Figure 1A**). The 5’ cap is important for translation initiation, through binding to the cap recognition protein eIF4E, which is engaged in the preinitiation complex eIF4F, facilitating ribosome binding and initiation of translation (9). The 2’ O-methylation of the cap (cap-1) is a characteristic of endogenous mRNA, allowing discrimination of self vs exogenous RNA (such as viral RNA), preventing inappropriate immune activation through detection by pattern recognition receptors (PRRs) (10). Indeed, this detection induces type I interferon (IFN) production and IFN-dependent signalling pathways that degrade and block the translation of RNA lacking 2′O methylation (cap-0), thus preventing translation of viral RNA (11). The poly(A) tail results from a nuclear polyadenylation, and its length is tightly regulated both in the nucleus and cytoplasm, notably through binding to the polyadenylate-binding protein (PABP), which plays dual functions: protecting and stabilizing the poly(A) tail, but also facilitating the deadenylation process through recruitment of deadenylases, under certain conditions that are not fully elucidated (12). Beside its importance for the protection and stability of mRNA, the poly(A) tail, in combination with PABP, participates in the stabilisation of the 5’ cap binding to the translation initiation complex, which is an important target of translation initiation regulation (8, 12, 13). While it has long been postulated that a positive correlation exists between poly(A) tail length and mRNA stability, recent findings also suggest that short poly(A) tails are a feature of highly expressed mRNA, while longer tails are associated with transcripts of lower abundance and poor translation (12, 14).

Of note, beside conventional mRNA vaccines, another type of mRNA, self-amplifying mRNA (saRNA), has been widely studied as a vaccine platform against infectious diseases, particularly for viral infections (15–20). saRNA contains a replicase gene derived from a viral replicon, usually from alphaviruses or flaviviruses, conferring them an auto-replicative activity. This results in higher antigen production in host cells, allowing to lower the dose of mRNA needed for vaccination and thus lowering the manufacturing costs (15, 21).

### *In vitro* synthesis of mRNA and optimization for vaccine development

mRNA vaccine production relies entirely on an *in vitro*, cell-free process, and is thus safe, quick and can be easily standardised (**Figure 1B**). It necessitates a DNA template (usually a linearized plasmid), in which the antigen and promoter sequences are encoded. *In vitro* transcription (IVT) of mRNA is then performed using an RNA polymerase from bacteriophage origin (such as T7, T3 or SP6) and nucleotides triphosphates (NTPs) under the control of the promoter, which is located upstream of the antigen sequence in the DNA template. Virtually any antigen sequence or multiple antigens can be transcribed with this method, and the DNA template can encode all the elements of functional mRNA except the 5’ cap, which needs to be added during the manufacturing process (6). Two methods are used for this purpose: a synthetic cap analogue (such as the CleanCap developed by TriLink) added during the IVT reaction to be directly incorporated in the synthesised mRNA, or alternatively a capping enzyme, such as the vaccinia capping enzyme, which will add the cap during an additional step after the transcription, in the presence of a methyl donor substrate. The poly(A) tail can either be encoded directly in the DNA template sequence or be added enzymatically with the recombinant poly(A) polymerase after IVT. However, this enzymatic process generates mRNAs with poly(A) tails of different lengths and thus direct encoding of poly(A) tail is often preferred for clinical applications (22).

mRNA instability, as well as its recognition by the innate immune system, are major obstacles for their use in therapeutic applications. Thus, several methods have been developed to increase the stability, translation efficiency and immune profile of therapeutic mRNA, such as codon optimisation, nucleotide modification, and selection of efficient purification processes (6). The codon optimization, consisting of the replacement of rarely used codons by synonymous, frequently used codons in humans, is commonly performed in order to increase the translation efficiency of the mRNA in the target cells. Indeed, due to species-specific differences in codon usage and abundance of transfer RNAs (tRNAs), which transport the corresponding amino acids to the ribosomes, this approach may increase the protein elongation rate, yet the importance of codon optimisation for therapeutic mRNA design is still debated as it may affect mRNA secondary structure, translation dynamics and protein conformation (23, 24).

Nucleoside modification, notably the replacement of uridines by pseudouridine and derivatives, has been widely used as a strategy to avoid recognition by innate immune receptors (detailed below), thus decreasing unwanted immune activation and side effects. Preclinical studies demonstrated that nucleoside-modified mRNAs induced increased translation efficiency, prolonged detectability, and increased immunogenicity of mRNA vaccines in animal models (25–27). Pfizer-BioNTech and Moderna have both adopted this strategy, as uridines are replaced by N1-methylpseudouridine in both licensed mRNA-based COVID-19 vaccines, BNT162b2 and mRNA-1273. Of note, while CureVac has always defended the interest of using non-modified mRNA despite the mitigated results of its COVID-19 candidate vaccine CVnCoV, they recently reported preliminary data of a study comparing two next generation nucleoside-modified versus unmodified COVID-19 mRNA vaccines (28). The results indicate that the modified mRNA induces similar antibody levels than the unmodified vaccine at the same dose, but with many fewer side effects, allowing to safely increase the dosage to achieve maximal protection efficacy. As a consequence, CureVac announced they will now focus their development activity on high dose modified mRNA vaccines for both its COVID-19 and flu vaccine programmes (28).

Regulatory regions in the 5’ and 3’ UTRs can also be modulated to increase mRNA stability and translation efficiency (29). For example, the COVID-19 vaccine BNT162b2 (Pfizer-BioNTech) incorporates the 5′-UTR of the highly expressed human α-globin gene, and the 3’-UTR contains regulatory elements of the α- and β-globin genes as well as other segments from the human mitochondrial 12S rRNA and *AES/TLE5* gene (30). Interestingly, a recent study in rhesus macaques comparing the immunogenicity and efficacy of the LNP-mRNA vaccine candidate CVnCoV (Curevac) and a similar construct with optimized UTRs (CV2CoV), showed that the CV2CoV elicited higher neutralising antibodies titres and memory B and T cell responses, correlating with higher protective efficacy than CVnCoV for the same dose, highlighting the importance of UTRs optimisation for vaccine design (31).

The purity of mRNA is essential for its therapeutic use, as DNA template, enzymes, residual NTPs or dsRNA contaminants generated during IVT can significantly impact on the translation efficiency and immunogenicity profile of the vaccine, due to innate immune activation (5). The choice of the purification methods depends on the production scale and application (i.e from laboratory research and clinical trials to mass industrial production of licensed products). At the laboratory scale, frequently used methods include DNase treatment, lithium-chloride (LiCL) precipitation, cellulose-based chromatography or the use of commercially available purification kits (32, 33). For large scale, GMP-compliant production, the purification usually relies on advanced chromatography technics, such as ion pair reverse phase chromatography (IPC), ion exchange chromatography (IEC), diafiltration using tangential flow filtration (TFF) and affinity chromatography, which are more efficient in removing short abortive mRNA and dsRNA (5, 34).

### Delivery of mRNA vaccines

mRNA vaccines need to reach the target cells and cross the cell membrane to enter the cytosol, where they use the cell machinery to be translated into the encoded antigen. However, naked mRNA is rapidly degraded by RNases in the extracellular space, and its negative charge prevents cell membrane crossing due to electrostatic repulsion. Thus, one of the main challenges is the development of efficient delivery systems to carry mRNA to the cytosol, while protecting it from degradation. Lipid-based carriers, such as liposomes or lipid nanoparticles (LNPs) are the most widely used for delivery of mRNA vaccines, but several other systems have been explored: polymers and polyplexes, such as polyethylenimine (PEI) or poly(amidoamine)s, which form complexes with nucleic acids and efficiently deliver mRNA to the cytoplasm, but can present a significant toxicity due to their positive charge; pH-responsive polymers, protonated at acidic pH in endosomes and presenting a lower toxicity profile; peptides and cell-penetrating peptides (CPPs) such as protamine that electrostatically bind to mRNA and form nanocomplexes due to their cationic or amphipathic amine groups; or squalene-based cationic nanoemulsions that adsorb mRNA on their surface (5, 22, 35).

Cationic liposomes such as N-[1-(2,3-dioleoyloxy)propyl]-N,N,N-trimethylammonium (DOTMA) and 1,2-dioleoyloxy-3-trimethylammoniumpropane (DOTAP) have been used successfully for *in vitro* transfection of mRNA to mammalian cells (Malone et al., (36); Zohra et al., (37)). However, the positively charged cationic lipids tend to aggregate with negatively charges serum proteins, increasing the clearance rate, and have a cytotoxic effect, limiting their clinical applications (35, 38). Incorporation of hydrophilic polymers such as polyethylene glycol (PEG) reduces the toxicity of cationic liposomes but can generate anti-PEG antibodies upon repeated administrations, increasing their clearance rate (39).

LNPs are among the most efficient and widely used mRNA delivery systems, and notably enter in the formulation of the COVID-19 vaccines developed by Moderna and Pfizer/BioNTech. LNPs are typically composed of cationic or ionisable lipids, and structural lipids including sterols, helper phospholipids and PEGylated lipids, and encapsulate mRNA in their core (35, 40). Various parameters influence LNP properties, and depend on the nature and proportion of the different lipids in LNP formulation: the size impacts on biodistribution and internalisation (with an optimal size usually comprised between 20 and 200 nm), the charge is important for cell uptake, cytotoxicity, encapsulation efficiency and organ targeting, while membrane hydration affects the fluidity, deformability, membrane fusion and responsiveness to pH, which is important for mRNA release in the acidic lysosome environment. Cationic and ionisable lipids are composed of 3 parts: the positively charged headgroup entraps the nucleic acid, interacts with the cell membrane and facilitates endosomal escape; the hydrophobic tails (typically 1 to 4) are saturated or unsaturated and impact the lipophilicity, fluidity and fusogenicity of LNPs; and the linker (composed of esters, amides or thiols), impacts on the stability, biodegradability, cytotoxicity and transfection efficiency of LNPs (40). Ionisable lipids are preferred over cationic lipids, as they have a neutral charge in biologic fluids and acquire their positive charge only at acidic pH, i.e in endolysosomes, while the constant positive charge of cationic lipids can lead to cytotoxicity by causing a destabilization of the cell membrane (38). Phospholipids, such as the 1,2-Distearoyl-sn-glycero-3-phosphocholine (DSPC) used in COVID-19 vaccines or the 1,2-Dioleoyl-sn-glycero-3-phosphoethanolamine (DOPE)) are located at the periphery of LNP and organise into a bilayer structure, influencing the fluidity, membrane fusion, as well as the biodistribution of LNPs, as they can be designed for organ-specific targeting (41–43). Sterols (such as cholesterol, oxidised cholesterol derivatives and phytosterols) are the most abundant lipids in LNP formulation (20-50% of total lipids). They are essential for surface organisation through stabilisation of the lipid bilayer, shielding the positive charge, and influence the fluidity of the nanoparticles, protein fusion and endosomal escape (44, 45). PEG-anchored lipids (2-5% of total lipids) are composed of the hydrophilic polyethylene glycol conjugated to an anchoring phospholipid. The amount, length and type of PEG-lipids regulate the size of LNPs by limiting lipid diffusion and protect them from aggregation and undesired interactions with biological environments, such as serum protein and macrophage phagocytosis. However, an excess of PEG lipids can reduce receptor-mediated cell uptake and transfection of LNPs, thus reducing mRNA delivery. This can be overcome by using a PEGylating agent with weak bilayer anchor or a cleavable linker, resulting in a progressive loss of PEGylation in biologic fluids (35, 43). They can also be used to functionalise the surface of LNPs to enable bioconjugation with ligands and macromolecules, for organ/tumour targeting for example (46).

## Immunological bases of mRNA vaccines

### Innate immune detection of exogenous RNAs

**Figure 2:**
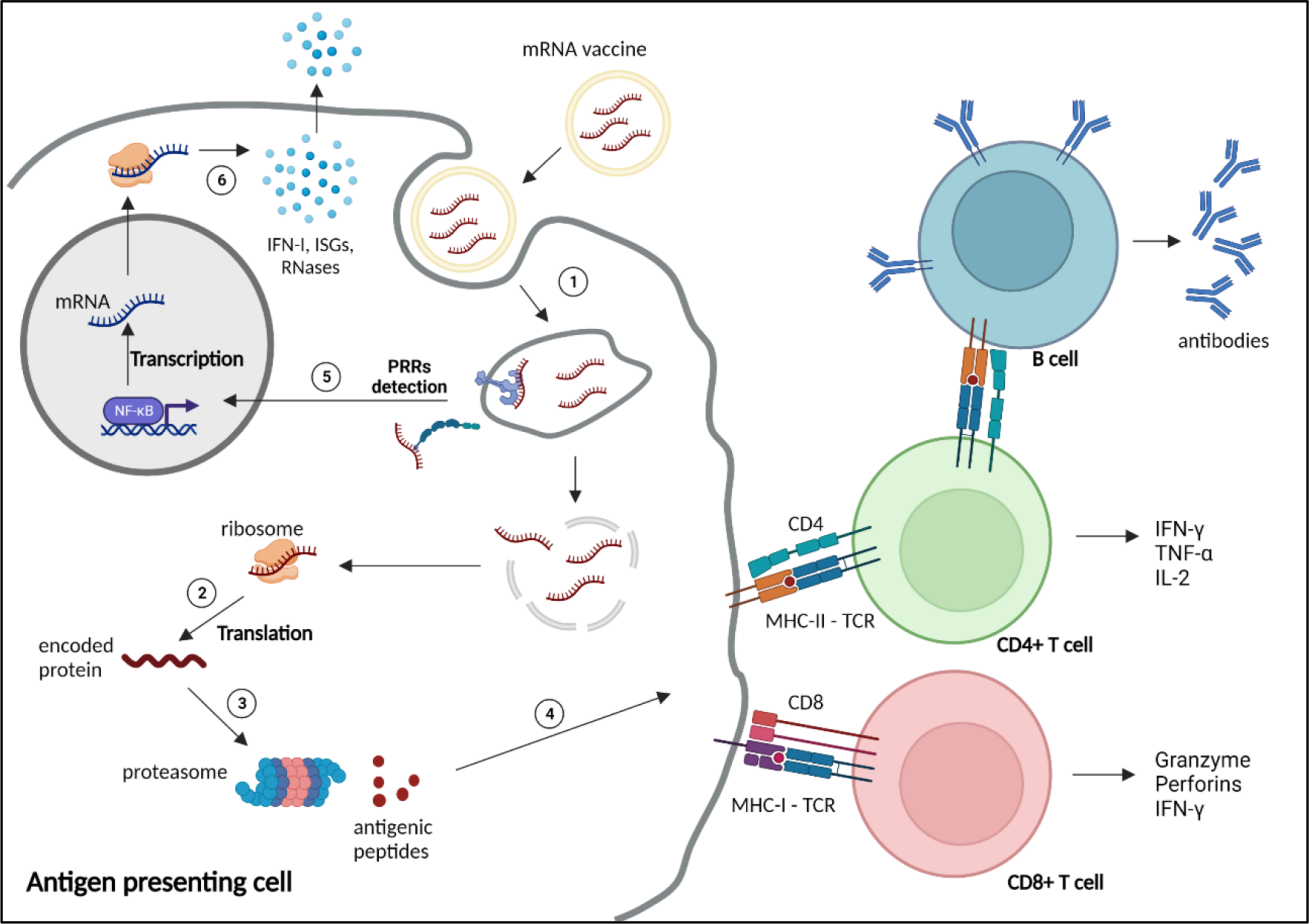
Mechanism of action and immune response induced by mRNA vaccines. 1) mRNA vaccines enter the cells through different mechanisms depending on the nature and size of the nanoparticles, such as clathrin-, caveolin- and receptor-mediated endocytosis, micropinocytosis, phagocytosis or diffusion across the cell membrane (47). 2) After reaching the cytoplasm, mRNAs are translated by the ribosomes into the encoded protein. 3) The protein is processed by the proteasome into small antigenic peptides. 4) The peptides are presented at the surface of the antigen presenting cell by major histocompatibility complex (MHC) molecules to prime CD4+ and CD8+ T cells through, respectively, MHC-II or MHC-I interaction with the T cell receptor (TCR), to activate humoral and cellular adaptive responses. 5) Exogenous mRNAs can be detected by the innate immune system through binding to pattern recognition receptors (PRRs) localised at the endosomal membrane or in the cytosol, inducing the transcription and translation (6) of proinflammatory factors, such as type 1 interferons (IFN-I), IFN-stimulated genes (ISGs) and RNases. NF-κB, nuclear factor κB. Created with BioRender.com.

Exogenous nucleic acids are detected by the innate immune system, as a coevolutionary mechanism of eucaryotes against invading pathogens. Exogenous RNAs are recognised by Immune-sensing receptors localised at the cellular or endolysosomal membrane, such as toll-like receptor (TLR)3, TLR7 and TLR8, or by cytoplasmic receptors, including the Retinoic Acid Inducible Gene-I (RIG-I), melanoma differentiation-associated protein 5 (MDA5) or nucleotide-binding oligomerization domain (NOD)-like receptors (NLR) Family Pyrin Domain Containing 1 (NLRP1). Activation of these receptors induces expression of pro-inflammatory genes, particularly type-I and type III IFNs, and IFN-stimulatory genes (ISGs), triggering various defence mechanisms to eliminate the pathogens (**Figure 2**). Additional mechanisms, such as antiviral restriction factors and RNases participate in exogenous RNA neutralisation and degradation (48). RNA degradation by RNases, such as RNase T2 and RNase 2, which occurs at sites containing uridine, generates by-products, that are further detected by TLR8, amplifying the immune response (49–51). Indeed, TLR7 and TLR8 have been shown to recognise guanosine- and uridine-rich ssRNA (51–53). Activation of this receptors induce cytokine production, particularly type I IFN, which promotes expression of RNA sensors, leading to a reduction of mRNA translation and increase inflammation (54). Thus, as previously mentioned, mRNA vaccine design often includes uridine replacement by methylpseudouridine, in order to avoid this mechanism.

The cap is also important in this regard, as the 5’-triphosphate end of RNA (m (7)GpppN, cap-0) can be detected by RIG-I, triggering type I IFN immune response, whereas N1 methylation of the 5’ cap (m (7)GpppNm, cap-1), which is a feature of self-RNA, prevents this recognition (55, 56). Similarly, the human IFN-induced protein with tetratricopeptide repeats 1 protein (IFIT1) binds to Cap-0, competing with the translation initiation factor complex eIF4F, thus reducing mRNA translation (57–59).

In addition, IVT mRNA production can generate double-stranded (ds) RNA by-products during the synthesis process, which can drive inflammatory and translation inhibitory pathways through TLR3, RIG-I, or MDA5 activation, as well as through detection by restriction factors such as the Protein kinase R (PKR), Oligoadenylate synthetase (OAS), and Adenosine deaminase acting on RNA (ADAR1) (60).

On the other hand, the production of inflammatory cytokines in antigen presenting cells (APCs) participates in the activation of adaptive immune response through enhancement of antigen presentation to T cells (61). These mechanisms are important considerations when designing and producing mRNA vaccines, and several optimisation and purification methods have been developed to limit the innate immune recognition to generate a balanced innate response, for efficient activation of APCs, while limiting mRNA degradation which reduces translation efficiency (5, 32, 61). In addition, high innate immune activation is responsible of acute post-vaccination symptoms, including injection-site pain, fever, chill, headache and fatigue, limiting the maximal dose that can be administered (62–64).

### Immunogenicity of mRNA vaccines and correlates of protection

There are several immunologic aspects to consider when developing a vaccine: the innate immune stimulation or “adjuvant effect”, and the adaptive response, which comprises humoral and cellular responses, as well as the generation of memory cells. The adjuvant effect is essential to the recruitment and activation of APCs and priming of T cells to induce adaptive responses. Therefore, it is often necessary to add adjuvant molecules to the vaccine formulation in order to increase its immunogenicity, particularly in the case of subunit vaccines (5). The humoral and cellular adaptive responses, relying on antibody production and cytotoxic T lymphocytes (CTL) respectively, are the two main effector mechanisms of acquired, -natural or vaccine-induced-, protection against pathogens. While live attenuated vaccines stimulate both responses, inactivated and subunit vaccines, although having a better stability and safety profile, often induce only humoral response (5). This is of importance since, while antibody titres are usually the main correlate of protection for most available vaccines (65), cellular immunity may participate to the protection against intracellular pathogens such as *M. tuberculosis (* 66–71) or influenza (72–74). Finally, as antibody titres fade over time after immunisation, the generation of long-lasting memory B cells and T cells, which can rapidly respond, upon new encounter with the pathogen, by clonal expansion and massive antibody production, is crucial for long-term vaccinal protection, particularly for diseases with long incubation time (65).

Since the first demonstration by Wolff and colleagues in 1990 of the ability of mRNA to induce protein expression after direct *in vivo* administration (75), several studies reported the production of specific antibodies and induction of CTL-mediated cellular immunity, highlighting its potential for vaccination against infectious diseases as well as for targeted cancer therapy (27, 76–80) (**Figure 2**). However, our understanding of the exact contribution of these mechanisms to the protection against infection is still incomplete and pathogen-dependent (54).

The innate response and the role of APCs have been investigated in a recent preclinical study with the nucleoside-modified mRNA-LNP COVID-19 vaccine BNT162b2 (Pfizer/BioNTech). The authors reported an increased activation of monocytes, macrophages and DCs in dLN, lung and spleen as well as increased cytokine and chemokine levels (including MCP1, Mip1b, CXCL10, IL-6, IFN-α and IFN-γ) in serum in the first hours/days following intramuscular vaccination (81). Consistent with observations in vaccinated patients (82, 83), this innate response was enhanced in the mouse model following the boost dose compared to the first dose, and was accompanied by a higher magnitude of IFN-γ-secreting CD4^+^ and CD8^+^ T cells in draining lymph nodes (dLNs), lung and spleen. By contrast, NK cells appeared to be the major source of IFN-γ after the first dose (81). Similar observations were made with an LNP-mRNA vaccine coding for influenza haemagglutinin H10 in rhesus macaques, with rapid and transient infiltration of neutrophils, monocytes and DCs, associated with type I IFN-inducible gene stimulation in dLNs, which correlated with priming of specific CD4+ T cells (84). However, while particle uptake by DCs and monocytes/macrophages resulted in high transcript translation, poor protein production is observed in neutrophils, suggesting that those cells may rather have a competitive role for particle uptake by APCs. In line with this, a study showed that, following intradermal administration of influenza vaccine, DC and langherans cells contribute to optimal T cell activation in dLNs, while neutrophils are dispensable (85). Of note, transgenic mice were used to demonstrate that MDA5 and Interferon-alpha/beta receptor alpha chain (IFNAR1) (but not TLR2,3,4,5,7, inflammasome or STING-cGAS) signalling pathways are essential for innate immune activation and induction of specific CD8^+^ T cell response following BNT162b2 vaccination, but have only a modest role in inducing specific antibodies, as IgG titres were only mildly decreased in deficient mice (81).

LNP-mRNA vaccines against SARS-Cov-2, HIV, influenza or Zika were shown to elicit durable antibody and Th1 cell responses in animals, associated with strong activation of antigen-specific T follicular helper (Tfh) and germinal centre B cells in dLNs (27, 86–89). The central role of rapidly -induced CD4+ T cells in orchestrating humoral and cellular responses during a prime-boost vaccination protocol against SARS-CoV-2 was highlighted in a longitudinal cohort analysis, where CD4+ T cells induced by the prime vaccination correlated with CD8+ T cells and antibody titres induced following the second dose in naïve healthy individuals (83). In addition, the prime-boost vaccination protocol with SARS-CoV-2 vaccines induces durable memory B cells and memory CD4+ and CD8+ T cells (detectable 6 months post-immunisation), similar to those induced by SARS-CoV-2 natural infection (83, 90, 91).

A large body of evidence points towards collaborative roles of humoral and CD8+ T cell-mediated responses in the protection induced by mRNA COVID-19 vaccines. In a phase 3 clinical trial, the titres of binding and neutralising antibodies against the viral spike protein were indeed correlated with protection against COVID-19 disease in individuals vaccinated with mRNA-1273 vaccine (Moderna) (92). The protective and therapeutic role of neutralising antibodies was further demonstrated in a study showing that the adoptive transfer of IgG from convalescent rhesus macaques to naïve animals protected recipient animals against SARS-CoV-2 intranasal challenge in a dose-dependent manner (93). Beside this, CD8+ T cell depletion partially abrogated the protection against rechallenge with SARS-CoV-2 in convalescent macaques with declining antibody titres (4 to 7 weeks post-primary infection (93). Another study points out a potential role of spike-specific CD8+ T cell response in early protection following prime vaccination with bnt162b2, when neutralising antibodies are hardly detectable (94). Interestingly, some observations suggest that memory T cells are less affected by immune escape observed with mutation variants of the virus than antibody-mediated responses and may thus be important for protection against SARS-CoV-2 variants (95, 96).

Of note, it is now recognised that LNPs, which enter in the composition of the commercialised COVID-19 vaccines and are the most widely used carriers in mRNA vaccine development, exert a strong adjuvant activity. LNPs containing ionizable lipids (iLNPs) were shown to induce a strong chemokine and cytokine production (including IFN-α, IL-6, IL-1b and GM-CSF), immune cell infiltration at the injection site and in dLNs, and promote DC maturation and monocytes and DC activation (27, 85, 97, 98). Moreover, iLNPs were shown to play an essential role in the stimulation of Tfh cells and germinal centre B cells, inducing their differentiation into long lived plasma cells and memory B cells, associated with a durable protective antibody response. This effect was mediated by IL-6 signalling pathway, and dependant on ionisable lipids (97). iLNPs have thus been successfully used as adjuvants in sub-unit vaccines against dengue and hepatitis B, VZV, influenza and SARS-CoV-2 (97, 99–101). In addition, several studies demonstrated that cationic lipid-based nanoparticles are detected by immune sensors, such as TLR4, TLR2, TLR3, NLRP3 or STING and enhance vaccine immunogenicity (102–105). IL-1 and IL-1ra are strongly produced by immune cells *in vitro* and *in vivo* upon administration of liposome-encapsulated mRNA vaccine and are important regulators of the inflammatory response induced by the vaccine in a lipid-dependent manner (106). Overall, these studies bring some clarification about the key innate pathways implicated in the induction of protective, specific immunity by mRNA-LNPs vaccines, even though complementary analyses are still required to better understand the contribution of memory B and T cells, particularly regarding strain-diversity and protection against reinfection with different variants in the case of SARS-CoV-2.

## Pre-clinical and clinical development of mRNA vaccines against infectious diseases

If the research on mRNA vaccine against infectious diseases have truly exploded since 2020, driven by the success of COVID-19 vaccines, first preclinical evidence of the potential of RNA-based vaccines for tumours and infections have emerged since the 90s. In 1993, mice immunization with liposome-encapsulated IVT mRNA coding for the influenza virus nucleoprotein was shown to induce virus-specific CTL (107). Of note, many of early preclinical reports on the induction of protective immunity against infectious diseases were conducted with self-amplifying RNA replicons derived from alphaviruses or flaviviruses, packed in virus-like particles (108). They targeted mostly viruses, such as influenza (16, 109, 110), vaccinia virus (111), parainfluenza virus type 3 (20), tick-borne encephalitis virus (16, 112), HIV (19, 113, 114), herpes simplex virus (115), or Ebola (18, 116). However, viral vectors present a risk due to anti-vector immunity, have limited loading capacity, and their production is more fastidious (108). Nonetheless, the undeniable advantages of mRNA over other vaccine platforms as a safe, highly adaptable and easily produced template for *in vivo* protein expression, with intrinsic adjuvant properties and no risk of insertional mutagenesis (unlike DNA), kept the interest of the scientific community despite the absence of clinical validation.

The use of lipid-based nanocarriers, formulated to protect and deliver small molecules such as nucleic acids to the cells, have significantly contributed to the mRNA vaccine field (40, 97). In parallel, important advances have been made in the design of mRNA, with modifications by genetic engineering to improve their stability, translation efficiency and immunogenicity/safety profile (117). These efforts culminated in the development and approval of two COVID-19 vaccines in a record time. As a result of this success, modified or unmodified mRNA formulated with LNPs are now predominant in both preclinical and clinical development of mRNA-based vaccines against infectious diseases (40) (**Table 1**).

**Table 1:** list of clinical trials evaluating mRNA vaccines against infectious diseases.

Application	Designation (Sponsor)	NCT number	Development phase	Date	Status
**Influenza**	mRNA NA vaccine (Sanofi Pasteur)	NCT05426174	Phase 1	2022	Active
	GSK4382276A (GSK)	NCT05446740	Phase 1	2022	Recruiting
	mRNA-1010 (Moderna)	NCT04956575	Phase 1/2	2021	Completed
		NCT05415462	Phase 3	2022	Active
	mRNA-1020 & 1030 (Moderna)	NCT05333289	Phase 1/2	2022	Completed
	MRT5407 (Sanofi Pasteur)	NCT05553301	Phase 1	2022	Recruiting
	Modified mRNA vaccine (Pfizer)	NCT05052697	Phase 1/2	2022	Recruiting
	Quadrivalent influenza modRNA vaccine (qIRV) (Pfizer)	NCT05540522	Phase 3	2022	Recruiting
	Quadrivalent Influenza mRNA Vaccine CVSQIV (CureVac)	NCT05252338	Phase 1	20222	Recruiting
	PF-07852352 & others Influenza saRNA (Pfizer)	NCT05227001	Phase 1	2022	Recruiting
**SARS-CoV-2 and Influenza**	mRNA-1073 (Moderna)	NCT05375838	Phase 1/2	2022	Active
**HIV**	eOD-GT8 60mer (mRNA-1644) (IAVI, Moderna)	NCT05414786	Phase 1	2022	Active
	mRNA-1644 & 1644v2-core (IAVI, Moderna)	NCT05001373	Phase 1	2021	Active
	BG505 MD39.3, BG505 MD39.3 gp151, and BG505 MD39.3 gp151 CD4KO HIV Trimer mRNA Vaccines	NCT05217641	Phase 1	2022	Active
**Zika**	mRNA-1325 (Moderna)	NCT03014089	Phase 1	2017-2019	Completed
	mRNA-1893 (Moderna)	NCT04917861	Phase 2	2021	Active
		NCT04064905	Phase 1	2019-2021	Completed
**CMV**	mRNA-1647 (Moderna)	NCT05085366	Phase 3	2021	Recruiting
		NCT05105048	Phase 1	2021	Recruiting
		NCT04975893	Phase 2	2021	Enrolling by invitation
		NCT04232280	Phase 2	2020	Active
		NCT03382405	Phase 1	2017-2021	Completed
**EBV**	mRNA-1189 (Moderna)	NCT05164094	Phase 1	2021	Recruiting
**RSV**	mRNA-1345 (Moderna)	NCT05127434	Phase 2/3	2021	Recruiting
		NCT05330975	Phase 3	2022	Recruiting
		NCT04528719	Phase 1	2020	Active
**hMPV, PIV3**	mRNA-1653 (Moderna)	NCT03392389	Phase 1	2018-2020	Completed
		NCT04144348	Phase 1b	2019	Active
**Rabies**	CV7201 (Curevac)	NCT02241135	Phase 1	2014-2018	Completed
	CV7202 (CureVac)	NCT03713086	Phase 1	2018-2021	Completed
**HSV**	BNT163 (BioNTech)	NCT05432583	Phase 1	2022	Recruiting
**TB**	BNT164a1 & BNT164 (BioNtech)	NCT05547464	Phase 1	2022	Not yet recruiting
		NCT05537038	Phase 1a	2022	Not yet recruiting
**Nipah virus**	mRNA-1215 (Moderna)	NCT05398796	Phase 1	2022	Recruiting
**Chikungunya virus**	mRNA-1944 (Moderna)	NCT03829384	Phase 1	2019-2021	Completed
**Malaria**	BNT165b1 (BioNtech)	NCT05581641	Phase 1	2022	Recruiting

Influenza vaccine research, for example, have largely beneficiated from these technologic advances. Indeed, the high mutation rate of Influenza viruses means that the vaccine formulation needs to be constantly adapted to the last circulating strains, which is difficult to manage due to the long production time of conventional influenza vaccines, and results in variable effectiveness. mRNA technology could help in improving antigen design, or ideally developing a universal, cross-reactive vaccine, as well as ease the production to better respond to seasonal epidemics and pandemics. Preclinical studies showed that multitargeting mRNA-LNP vaccines elicit broad protective immunity in animal models against multiple Influenza virus strains (118–122). Several influenza vaccines have reached clinical trials, and Moderna (mRNA-1010) and Pfizer (qIRV) vaccine candidates have recently entered phase 3 of clinical trials (**Table 1**).

In recent years, nucleotide-modified or unmodified IVT mRNA vaccines formulated with lipid-based vectors have demonstrated efficacy in animal models against various infectious diseases, such as RSV (123), rabbies (80, 124), malaria (125), HIV (126–128), Ebola (129), Zika virus (89) and Cytomegalovirus (130). Clinical trials, active or completed, evaluating mRNA vaccines for infectious diseases, to the exclusion of COVID-19, are listed in **Table 1**. Of note, mRNA vaccines against RSV (mRNA-1345) and CMV (mRNA-1647), both developed by Moderna, have also entered phase 3 clinical trials.

## Interest of mRNA vaccine technology for TB, HIV and malaria

TB, malaria and acquired immunodeficiency syndrome (AIDS), remain among the leading causes of death by infectious diseases, particularly in low-income countries (WHO, top 10 causes of death). Despite considerable efforts, the development of safe, efficient, and cost-effective vaccine easily deployable in endemic areas of these diseases remains a global health priority. The path opened by COVID-19 mRNA vaccines thus raises a new hope for the fight against these infections.

### Tuberculosis

TB is caused by a pulmonary bacterium, *Mycobacterium tuberculosis* (M.tb). The disease classically progresses from latent TB infection (LTBI), where the bacteria are contained by the host immune system inside lung granulomas, to active TB disease if the pathogen is not eliminated and escapes immune containment. While reactivation of LTBI is the major source of TB disease patients, some patients develop active TB disease soon after M.tb exposure, within months or few years (131). During active TB, the patient is symptomatic (the main symptoms are fever, cough, weight loss, haemoptysis) and contagious. Of note, HIV infection is the strongest risk factor for TB disease, accounting for around 25% of all TB-related deaths (131). An antibiotic regimen against TB exists, which classically combines four molecules for several months, in order to target all populations of bacteria and avoid emergence of resistance. However, poor access to diagnostic, treatment, and healthcare support in the regions where TB is the most prevalent favours the emergence and rapid spread of antibiotic resistant M.tb strains, further aggravating the situation (Global Tuberculosis Report (132), WHO).

Thus, an efficient and cost-effective vaccination program appears as the best strategy to face this situation. Despite BCG being the oldest licensed vaccine still in use, with high coverage throughout the world, TB remains the second leading cause of death by an infectious disease after COVID-19, killing around 1.6 million people per year essentially in low and middle-income countries (132–134).

Macrophages, dendritic cells and T cells are implicated in the control of bacterial growth in granulomas, preventing the spread of bacteria in blood circulation and progression to active TB (131). Of note, HIV/AIDS, characterised by an impairment of CD4+ T cells, is a major risk factor for progression to active TB disease. Furthermore, the lung and spleen protection following vaccination of mice correlates with the magnitude and quality of multi-functional CD4 T cells expressing IFN-γ, TNF-α, and IL-2 (135). While the mechanisms of natural and BCG-induced protective immunity against mycobacteria are incompletely elucidated, this suggest a role for cellular immunity for the control of infection and for vaccine-induced protection against M.tb (136). Overall, the complexity of M.tb culture and difficulty to establish a relevant animal model for TB infection, as well as the lack of clearly defined correlates of protection both in preclinical animal models and in clinical trials, are still major challenges for researchers (131).

Mycobacteria are complex microorganisms, encoding about 4000 genes, and the identification of immunodominant and protective antigenic targets have been a major focus for the design of effective protein subunit or nucleic acid-based vaccine against TB, even though whole pathogen vaccines, attenuated or inactivated, and viral vector-based vaccines are also being investigated (137–139). Immunopeptidomics, based on mass spectrometry identification of MHC-bound peptides from infected cells, has been demonstrated as a useful approach for the identification of novel antigenic peptides for vaccine development (140). Furthermore, Immunoinformatic approaches provide useful tools for *in silico* modelling of mRNA vaccines against TB, combining epitope identification, mRNA construction and optimisation and immune simulation to guide further *in vivo* evaluation (141–143).

Interestingly, the first proof of concept of an mRNA vaccine against TB has been reported in 2004. The authors demonstrated that immunisation of mice with naked IVT mRNA coding for the immunodominant antigen MPT83 (four injections at 3-weeks intervals) induced specific humoral and cellular responses and conferred a modest but significant protection against TB challenge (144). Intranasal immunisation with naked mRNA coding for Hsp65 protein also demonstrated significant protection against M.tb in mouse model (145). More recently, a replicating mRNA-based vaccine coding for a fusion protein comprising 4 M.tb Antigens (Rv3619, Rv2389, Rv3478, Rv1886) formulated with a lipid nanocarrier induced cellular immune response and protection against M.tb and M. avium challenge in mice in a heterologous RNA-prime and protein-boost vaccination protocol (146, 147). Of note, a phase I clinical trial evaluating two investigational RNA-based vaccines against TB in BCG-vaccinated volunteers have been launched by BioNTech (**Table 1**).

### HIV

In 2021, 650 000 persons died from HIV/AIDS-related cause, 1.5 million were newly infected, while around 38.4 million people were living with HIV (www.unaids.org). In the absence of a licensed vaccine, the arsenal against HIV/AIDS still relies on information and screening campaigns and antiretroviral therapies (ART). Even though ART have been significantly improved over the years, with stronger efficacy and less side effects than in the past, they remain expensive and require strict observance. Thus, poor access to diagnostic and treatment in low and middle-income countries with high prevalence (particularly Sub-Saharan Africa) is a major issue (148). Despite the dedication of governments, health organisms and the scientific community, no vaccine against HIV has been licensed to date. Indeed, in the last decades, numerous vaccine candidates have reached phase III efficacy trials, but none of them demonstrated sufficient efficacy (149, 150).

The proposed correlates of protection, identified in animal models and clinical studies, are neutralizing antibodies targeting HIV envelope (Env) glycoprotein epitopes, preventing the entry of virions in CD4+ T cells, and antibody-dependent cellular cytotoxicity (ADCC) (relying on Fc-mediated effector functions of non-neutralising antibodies), to eliminate infected cells and prevent virus reactivation from reservoir cells (151–153). However, targeting the highly glycosylated Env glycoprotein is hampered by the high mutation rate of the virus, resulting in high viral diversity (pseudoviruses), while broad neutralizing B cell precursor development is difficult to elicit and limited by immune tolerance mechanisms such as auto- or polyreactivity (150, 152, 154). Thus, vaccine design needs to be optimized to target a combination of multiple conserved epitopes from Env glycoprotein, eliciting broad neutralizing antibodies, as well as T cell epitopes on other viral proteins, such as Gag, Pol and Nef, to elicit CD8+ T cell-mediated responses (148, 153, 155). mRNA technology, combined with efficient delivery system, may hold the potential to overcome the challenges faced in HIV vaccine development.

Recent preclinical studies of mRNA-based vaccines in mouse and non-human primate models are indeed encouraging. Intradermal immunization of rabbits and rhesus macaques with mRNA-lipid nanoparticle (mRNA-LNP) vaccines encoding the clade C transmitted/founder HIV-1 Env 1086C elicited high levels of gp120-specific antibodies, with significant but transient neutralising activity, as well as ADCC activity in serum (126). In macaques, an HIV-1 Env-encoding mRNA-LNP elicited comparable titres and functions of neutralising and non-neutralising antibody than adjuvanted Env recombinant protein (156). In addition, Immunisation with polymer-formulated self-amplifying mRNA encoding mosaic *Gag-Pol* epitopes induced potent specific CD8+ T cell response in mice (17). Furthermore, an mRNA-LNP vaccine encoding *Gag* conserved elements induced potent humoral and cellular responses in macaques in a prime-boost protocol with Gag DNA vaccine (128). Interestingly, the authors report that higher dose of mRNA vaccine increased T cell, but not humoral response. In another study, mRNA-LNP vaccine encoding HIV-1 Env and simian immunodeficiency virus (SIV) Gag proteins to generate virus-like particles, induced broad neutralising antibodies and reduced the risk of infection in rhesus macaques immunized through a prime-boosts protocol with autologous and mixed heterologous Env challenged with repeated low doses of heterologous SHIV (127).

The HIV mRNA vaccine eOD-GT8 60mer (mRNA-1644), developed by the International AIDS Vaccine Initiative (IAVI), in collaboration with Moderna, is in phase I clinical trial to evaluate the safety and immunogenicity in healthy adult volunteers (**Table 1**). This promising candidate vaccine was designed to target germinal centre’s naïve progenitor naive B cells to produce broad neutralising antibodies (press release, First-in-human clinical trial confirms novel HIV vaccine approach (iavi.org)). The National Institute of Allergy and Infectious Diseases (NIAID) is also evaluating the safety and immunogenicity of three HIV trimer mRNA vaccines (BG505 MD39.3, BG505 MD39.3 gp151, and BG505 MD39.3 gp151 CD4KO) in healthy individuals (**Table 1**).

### Malaria

Malaria, caused by *Plasmodium falciparum* parasites transmitted by female *anopheline* mosquitoes, is responsible for 200 million infections and 400 000 deaths per year, particularly in young infants in endemic areas, such as sub-Saharan Africa (157). Anti-malaria drugs, while effective to prevent and treat the infection, are poorly accessible to populations living in endemic areas in LMIC, and the progression of parasitic resistance to available molecules compromises their efficacy. In this context, the development of a cost-effective vaccine is a highly relevant strategy to reduce the global burden of the disease.

The parasite has a complex life cycle with multiple development stages in human and mosquitoes. The sexual cycle occurs in the mosquito, which transmits the sporozoites through the skin. Sporozoites then migrate to the liver, where they invade hepatocytes and develop into merozoites, that are released in the blood circulation and invade erythrocytes, causing repeated cycles of erythrocytes invasion and lysis, responsible for the fever characteristic of malaria. Some merozoites develop into gametocytes, which are transmitted to the mosquito during the blood meal.

One of the first vaccination strategies investigated, consisted of the administration of attenuated sporozoites, the infective form of the parasite (158). However, injection of purified, radiation-attenuated whole sporozoites (PfSPZ), despite excellent protection in non-human primates and rodents, as well as in naïve adults when administered intravenously (but not *via* subcutaneous or intramuscular route) (159, 160), failed to efficiently protect previously exposed adults and infants (161, 162).

The immune mechanisms underlying protection are not completely elucidated, but the role of liver CD8+ T cells have been demonstrated in animal models, and a role for γδT cell priming and Fc-dependent antibody effector functions have been suggested (157, 163). Of note, the circumsporozoite protein (CSP), expressed at the surface of sporozoites of different Plasmodium species, has been identified as a major immunogen eliciting binding antibodies that prevent infection of hepatocytes, leading to the development of vaccines targeting the CSP (164, 165). Among those, the adjuvanted subunit vaccine RTS,S/AS01 (Mosquirix™) developed by GSK, composed of CSP repeats (R) and C-terminal T-cell epitopes (T) recombinantly fused to HBsAg, demonstrated modest but significant protection against *P. falciparum* infection in children in endemic areas (but not in adults), and the World Health Organization (WHO) has recommended its widespread use among children living in malaria endemic areas (164). However, the protection wanes over time, correlating with decreased anti-CSP antibody levels, and the vaccine does not prevent the infection of mosquitoes by gametocytes from infected individuals, and thus does not decrease the circulation and transmission of the parasite (164). Of note, the next generation R21-matrix M vaccine, composed of a fusion of CPS and HBsAg that aggregates as virus-like particles and formulated with matrix M adjuvant, demonstrated 77% efficacy in children aged 5-17 months in a phase 1/2b clinical trial in Burkina Faso over 6 months after 3 doses, and a booster dose 1 year after initial vaccination allow to maintain high protection efficacy in those children (165–167). Despite these encouraging results, a malaria vaccine eliciting long-lasting, high-level protection in all subgroups and which could block the transmission in endemic areas has yet to be developed.

So far, preclinical studies on mRNA vaccines against malaria are still scarce. A mRNA-LNP vaccine coding for the major sporozoite-targeting antigen PfCSP demonstrated potent antibody and cytokine responses in mice, and protected the animals in a dose-dependent manner against *P. burghei* infection (168). In another study, the authors evaluated two mRNA-LNP vaccine candidates encoding PfCSP and Pfs25, a protein expressed by ookinetes, essential for oocyst development (125). These vaccines thus block different stages of the parasite cycle, i.e the liver stage (prevention of hepatocyte invasion by sporozoites), and sexual stage (disruption of sexual cycle and transmission by mosquitoes), respectively. Both vaccines were highly immunogenic following single or co-immunisation, eliciting high, dose-dependent antibody titres and B and T cell responses in mice. The Pfs25 mRNA-LNP elicited potent mosquito transmission-blocking activity, while PfCPS mRNA-LNP alone or in combination with Pfs25 mRNA-LNP conferred significant protection against sporozoite infection challenge (125). This study supports the evidence that multiple stage targeting mRNA vaccine platforms may provide an effective strategy to contribute to decrease malaria transmission. Additional potential antigenic targets for vaccines were identified in preclinical studies, such as the protein PfGARP, expressed on the exofacial surface of erythrocytes infected by early-to-late-trophozoite-stage parasites. Cohort studies on Tanzanian children and male adults showed that detection of naturally-acquired anti-PfGARP-A antibodies in the blood was associated with a significantly lower risk of severe malaria and lower parasitemia (169). Anti-PfGARP antibodies demonstrated potent parasite killing activity *in vitro*, and immunisation with PfGARP-A-mRNA LNPs of *Aotus* monkeys challenged with *P. falciparum* resulted in significantly lower parasitemia than control monkeys (169). The Cell-Traversal protein for Ookinetes and Sporozoites (CelTOS), a secreted protein playing a role both in mosquito transmission and hepatocyte invasion, has also been identified as a potent vaccine target in preclinical settings (170). The authors demonstrated that CelTOS-targeting mRNA vaccine can induce potent humoral and cellular response in mice and highlighted the importance of careful vaccine design. However, further investigations are required to state on the protective efficacy of this vaccine platform and the roles of humoral and cellular responses in the protection. Of note, a phase 1 clinical trial is evaluating the safety and immune response of an mRNA-based vaccine targeting PfCSP, developed by BioNTech (**Table 1**).

## Conclusions

The success of mRNA vaccines against COVD-19 inspired scientist throughout the world to develop new mRNA vaccines against transmissible and non-transmissible diseases. Twenty-seven mRNA vaccine candidates against infectious diseases are in clinical trials and hundreds are currently being developed in pre-clinical studies. The number of mRNA vaccine candidates in clinical trials is expected to increase in the coming years. Moreover, the mRNA vaccine technology is being improved with new discoveries and developments at a daily basis.

Many complex diseases, particularly those that depend heavily on T cells for protection, lack the identification of the most protective antigens and the corresponding correlates of protection. In recent years, immunopeptidomics has been applied to identify antigens presented by MHC-I and MHC-II in an unbiased way, leading to the identification of antigens for vaccine development (140, 171). The combination of the most relevant antigenic targets identified by immunopeptidomics with the simplicity of the mRNA vaccine technology, allows for the development of vaccines encoding multiple antigens, or antigens to prevent multiple diseases, and paves the way for a new generation of more efficacious and effective vaccines against some of the most dramatic infectious diseases that depend on T cells for protection, such as malaria, TB and HIV.

The challenges faced by the mRNA vaccine technology includes the cost of raw materials, the lack of standardization, and delivery optimization. The high cost of raw materials and the cold-chain requirements for mRNA vaccines since manufacture to the moment of vaccination, will have an important impact on the vaccine cost-effectiveness. Moreover, the mRNA purity can vary significantly between different purification processes, which may contribute to the lack of process standardization. Importantly, the Intellectual Property landscape and the difficulty of access to methods and technologies, particularly to reagents and manufacturing processes required for vaccine formulation development, may delay the progress of new mRNA vaccines against the deadliest hard-to-treat infectious diseases malaria, TB and HIV.

Thus, we should expect future mRNA vaccines designed and optimised to induce the right type of immune response against each disease for maximal efficacy, improved stability of formulations, as well as formulations adapted to long term storage at room temperature, leading to a reduction and eventual elimination of cold-chain supply, ease of distribution, and above all, reduced cost. Of particular relevance is the difficulty of equitable distribution of mRNA vaccines to LMICs and remote regions. To overcome this difficulty, BioNtech have implemented modular mRNA manufacturing facilities to produce vaccines locally, being already installed in Rwanda (press release, BioNTech Starts Construction of First mRNA Vaccine Manufacturing Facility in Africa | BioNTech).

The mRNA vaccines represent a new generation of vaccines against transmissible and non-transmissible diseases. We are witnessing a scientific revolution, with direct consequences to improving global health. The success of the mRNA vaccines will change the history of medicine.

## Author contributions

All authors listed have made a substantial, direct, and intellectual contribution to the work, and approved it for publication.
